# An Efficient Neural-Network-Based Microseismic Monitoring Platform for Hydraulic Fracture on an Edge Computing Architecture

**DOI:** 10.3390/s18061828

**Published:** 2018-06-05

**Authors:** Xiaopu Zhang, Jun Lin, Zubin Chen, Feng Sun, Xi Zhu, Gengfa Fang

**Affiliations:** 1 College of Instrumentation and Electrical Engineering, Jilin University, Changchun 130061, China; xpzhang16@mails.jlu.edu.cn (X.Z.); lin_jun@jlu.edu.cn (J.L.); czb@jlu.edu.cn (Z.C.); 2 Key Laboratory of Geophysical Exploration Equipment, Ministry of Education, Jilin University, Changchun 130061, China; 3 School of Electrical and Data Engineering, University of Technology Sydney, Sydney, NSW 2007, Australia; Xi.Zhu@uts.edu.au (X.Z.); gengfa.fang@uts.edu.au (G.F.)

**Keywords:** microseismic monitoring, event detection, edge computing, neural networks, probabilistic inference

## Abstract

Microseismic monitoring is one of the most critical technologies for hydraulic fracturing in oil and gas production. To detect events in an accurate and efficient way, there are two major challenges. One challenge is how to achieve high accuracy due to a poor signal-to-noise ratio (SNR). The other one is concerned with real-time data transmission. Taking these challenges into consideration, an edge-computing-based platform, namely Edge-to-Center LearnReduce, is presented in this work. The platform consists of a data center with many edge components. At the data center, a neural network model combined with convolutional neural network (CNN) and long short-term memory (LSTM) is designed and this model is trained by using previously obtained data. Once the model is fully trained, it is sent to edge components for events detection and data reduction. At each edge component, a probabilistic inference is added to the neural network model to improve its accuracy. Finally, the reduced data is delivered to the data center. Based on experiment results, a high detection accuracy (over 96%) with less transmitted data (about 90%) was achieved by using the proposed approach on a microseismic monitoring system. These results show that the platform can simultaneously improve the accuracy and efficiency of microseismic monitoring.

## 1. Introduction

**Figure 1:**
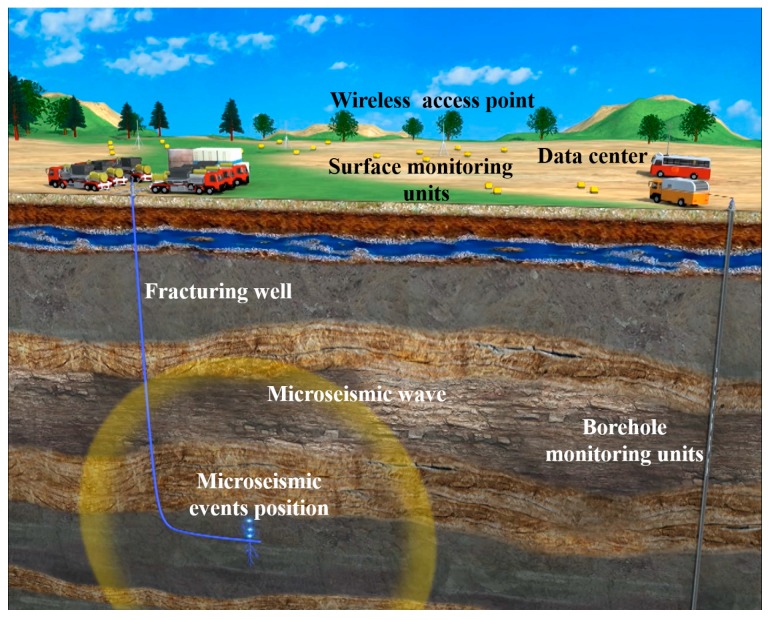
Microseismic monitor schematic diagram.

Hydraulic fracturing is a critical technology to improve oil and gas production that has been especially driven by the “shale gas revolution”, since the propagation paths in low-permeability reservoirs, where hydrocarbons may flow, are only created by hydraulic fracturing [1,2,3,4]. According to a report provided by the Energy Information Administration (EIA) in 2016, more than 50% of crude oil production in the U.S. was yielded by hydraulically fractured wells [5]. Hydraulic fracturing, as an essential technology for the development of unconventional resources, has also been used in over 2 million wells worldwide and about 90% of new U.S. gas wells [6]. Before hydraulic fracturing, people firstly shoot at the pipe of the fracturing well underground to create some initial cracks. Then, these cracks are fractured to form propagation paths. So, it is critical for hydraulic fracturing to be aware of the locations and growing trends of these cracks [7,8]. Usually, during the hydrofracturing process, a microseismic monitoring system is needed to map the locations of fractures by exactly sensing the induced microseismic events in order to, in a timely fashion, determine the correct fracture orientation and dimension to make the propagation paths grow efficiently [9]. As shown in Figure 1, a microseismic monitoring system mainly consists of a data center, surface monitoring units, and borehole monitoring units [10]. To monitor the changes of underground cracks as a correct reference for hydraulic fracturing, the data center collects and analyzes the data acquired by monitoring units. For providing the references to hydraulic fracturing in time, a microseismic monitoring system is required to perform at high accuracy in a real-time fashion. Therefore, the accuracy of microseismic event detection and the data collection time are two important indices of a microseismic monitoring system. In summary, to improve the efficiency of hydraulic fracturing, there are two major challenges in microseismic monitoring. The first challenge is how to identify the induced microseismic events with high accuracy. The second one is how to make the microseismic data collection as fast as possible so that the system can work in real-time.

In the last few decades, several solutions to achieve high-quality microseismic monitoring have been proposed as a consequence of the increasing demand for petroleum and natural gas worldwide. Most of them are mainly concerned with the accuracy of microseismic events detection [11,12,13,14,15]. On the other hand, for a real-time system, some solutions are presented to compromise accuracy for faster data transmission [16,17,18]. Until now, there is no such solution that can be used to support a high accuracy of events detection with a short data transmission time simultaneously. To address these two issues, we design a neural-network-based monitoring platform, named Edge-to-Center LearnReduce Microseismic Monitoring Platform, which could be used in a microseismic monitoring system, with an edge computing architecture. For improving the platform’s accuracy, a neural-network-based microseismic event detection model is developed, which is combined with a convolutional neural network (CNN) and long short-term memory (LSTM). The model’s parameters and structure are designed by considering the features of microseismic events. In addition, upon the neural network model, a probabilistic inference is applied to minimize false negative results. For the data transmission issue, an edge computing architecture is proposed based on the framework of a monitoring network so that the microseismic data needed to be transmitted from edge components to the data center can be dramatically reduced.

The rest of this paper is organized as follows. In Section 2, related works in three main approaches to monitoring microseismic events efficiently are reviewed and their drawbacks are identified. Then, our proposed efficient microseismic monitoring platform is presented in Section 3. In Section 4, both simulation and measurement results are presented and analyzed. Finally, the conclusion is given in Section 5.

## 2. Related Work

Until now, the research on microseismic monitoring has been mainly focused on detecting microseismic events. In this regard, the most extensively utilized method is the short-term average to long-term average (STA/LTA) algorithm, whose main idea is based on the differences in the energy densities of noise and a signal [13]. Based on this method, various varieties of STA/LTA methods have been published, including methods that consider situations with a high level of noise [12,19]. By calculating and considering some parameters of energy, amplitude, or other entropy functions in multiple windows instead of using average energies in a fixed-length window, the performance compared with that of the initial STA/LTA method can be significantly improved. However, these algorithms need a relatively long time to obtain the required accuracy due to the computational cost of parameters in different windows. Another widely used approach to recognize events is based on spectrum analyses of temporal, spatial, and frequency domains, such as a wavelet transform [20,21], a Hilbert–Huang transform [22], and other time-frequency representations [23]. The algorithms in this approach attempt to give insight into the complex structure of a noisy microseismic signal by displaying the amplitudes of different frequency components for a given time so that microseismic events can be separated from specified random noise. However, these methods are either not very effective for removing high-amplitude unknown noise or need some parameters to be tuned manually. In the past decade, with the fast development of artificial intelligence, machine learning techniques have been used for the development of microseismic event detection methods, such as the fuzzy clustering algorithm [14], support vector machine (SVM) [24], and the Bayesian probability model [25]. Although all of these learning algorithms can obtain good results for detecting microseismic events within a short computational time, the data collection time, referred to as the second challenge, is not considered in this category of solutions since they only pay attention to the data center and do not view the whole system as a platform.

Besides the studies on detecting microseismic events, some researchers are dedicated to compressing the microseismic data in the terminals, in an attempt to reduce the volume of data to be transmitted, so that the microseismic monitoring system can have less of a time delay in data retrieval [26,27]. However, this approach will add either some noise in fracturing mapping or extra computational time for data recovery in the data center. Therefore, methods that use compression can hardly solve the two challenges simultaneously.

In summary, the drawbacks mentioned above are caused due to the fact that the performance of each part of the monitoring system is optimized separately. Therefore, the aim of this work is to provide an efficient solution by taking into consideration the whole platform. The new platform consists of edge components and a data center. To achieve high accuracy for microseismic event detection, we use shoot data, which includes the main information of the underground structure and characteristics to train a specific model in the data center before hydraulic fracturing. After training, this learned model will be sent to an edge component for detection of microseismic events. To reduce traffic load, the edge component focuses on detecting microseismic events exactly and only transmits data including microseismic information.

## 3. Edge-to-Center LearnReduce Microseismic Monitoring Platform Design

### 3.1. Platform Structure

**Figure 2:**
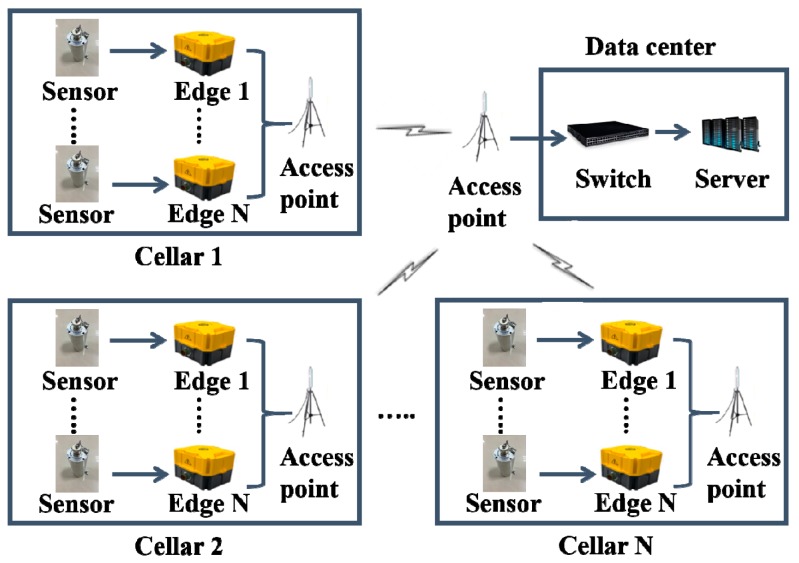
The structure of Edge-to-Center LearnReduce Microseismic Monitoring platform.

The structure of the Edge-to-Center LearnReduce Microseismic Monitoring Platform (ELMMP) is shown in Figure 2. The new platform consists of edge components and a data center. The edge components consist of the microseismic monitoring system’s surface monitoring units and borehole monitoring units, while the data center is the system’s data center. The data center and edge components are connected by access points. At an edge component, microseismic data is collected and processed by an embedded microchip and operation system. At the data center, a microseismic events detection model will be learned firstly by using shoot data. Then, the learned model will be sent to the edge components via access points to implement data reduction. After that, the data center uses the reduced data transmitted from the edge components to locate the source of a microseismic event in the fracturing.

When the platform is working, an edge component collects shoot data first and then transmits it to the data center for a learning purpose so that microseismic events can be detected exactly. Then, the edge component will download the learned microseismic events detection model from the data center before hydrofracturing work begins. While hydrofracturing, microseismic data is firstly recorded by the edge component. Secondly, the raw data is going to be pre-processed by some filters to reduce some ordinary noise. Thirdly, the data output by the pre-processing module will be used as the input of the neural network model that is downloaded from the data center, whose output can be considered as the possibility of a seismic event at each sampling point in the time domain. Then, a probabilistic inference, based on the probability result yielded by the neural network model, is used to output the minimum data only included by microseismic events. Finally, the edge component only transmits the data contained in microseismic events to the data center.

In the data center, the shoot data will be used to train a multiple-layer neural network model, at the beginning, using a strategy named end-to-end learning. This strategy is based on moving away from hand-crafted feature detectors. The model is trained to produce the probabilistic result of a microseismic event directly from the input data. To meet the training requirement, we first label the shoot data according to the shoot time. In the shoot data, 1 or 0 is used to describe whether there is a a microseismic event or not, respectively. Then, the shoot data and its label are imported to the neural network model proposed in this work, which comprises convolution and recurrent layers. It aims to extract both intrinsic features and temporal characteristics of microseismic events. After training, the learned model will be transmitted to the edge components.

### 3.2. The Data Center in ELMMP

#### 3.2.1. Overview of the Data Center Model

**Figure 3:**
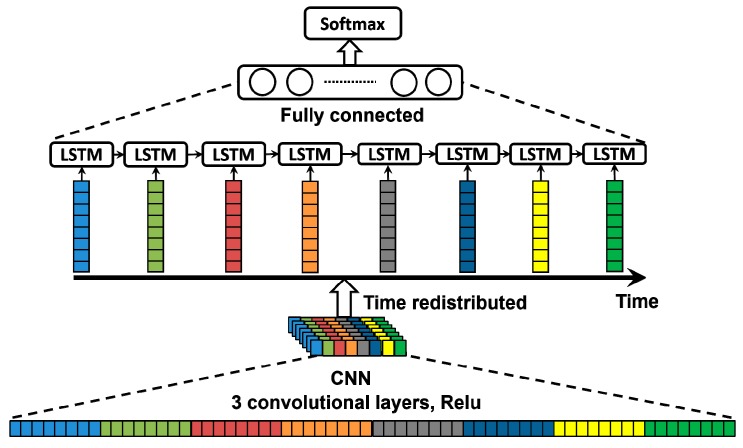
Architecture of the neural network for microseismic event detection. LSTM, long short-term memory; CNN, convolutional neural network.

These days, artificial neural networks, such as convolutional neural networks (CNNs) and recurrent neural networks (RNNs), have achieved state-of-the-art performance on several pattern recognition tasks, including image recognition [28,29,30]. Although prior knowledge of recognition will not be explicitly integrated into the neural network, it is important to give the network a structure that enables it to learn these dependencies from the data. Obviously, microseismic data detection is a kind of time-series data classification, which must depend on not only local features at the moment but also correlations with the past. Considering the applications of CNNs and RNNs to time-series classification, CNNs are attractive for their feature-learning ability but are not sensitive to the temporal characteristics of time-series data, while RNNs are effective for sequencing data but not good at capturing specific features locally. So, to obtain high accuracy in classification, we combined two networks to leverage the advantages of both CNNs and RNNs. To take the common local features of microseismic data from different sensors into account, the CNN module is used to capture the main local features of microseismic events in a specific hydrofracturing process. For detecting the temporal dependencies of these features, an RNN (LSTM) layer is used in our work, since recurrent connections can store memories of past features. Generally, for the microseismic event detection case, we design a neural network with a convolution module, a recurrent module, and a softmax layer as shown in Figure 3.

#### 3.2.2. Training Set

**Figure 4:**
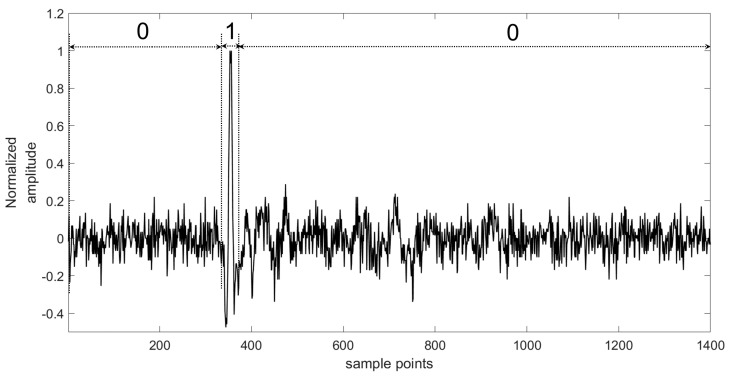
Shoot data and its label (training set).

As with traditional work in supervised learning, we must label the training data first. In this study, we use shoot data as training data and label it manually. Because of the high-level energy of a shoot event and awareness about the time crews have to shoot in the well, a microseismic event in the shoot data could be found out easily. Then, we could recognize a microseismic signal’s arrival time and duration time by analyzing the specific morphology features of the microseismic signal and seeking the shoot time. In Figure 4, there is an example of a piece of shoot data and its corresponding label sequence. The label sequence has the same length as the data sequence. It should be noted that the part of the label sequence marked “1” includes the arrival point and duration of the microseismic signal, and the remainder is marked as “0”.

#### 3.2.3. CNN Module

**Figure 5:**
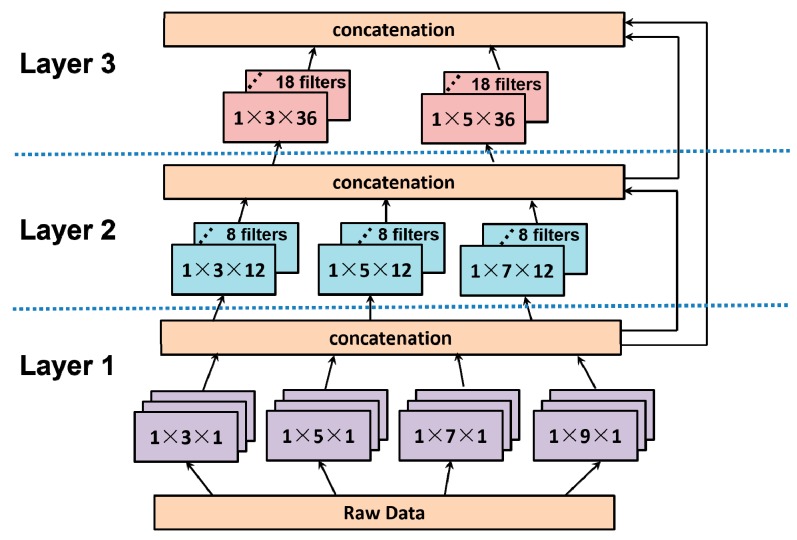
The convolution model in the designed neural network.

After the training set is labeled, the neural network begins to be trained. The convolution module is at bottom of the whole model. According to prior work in the study of the microseismic signal process, there are many different nonlinear features in microseismic events [31]. So, for improving the ability to capture the main characteristics of microseismic events, we design a convolution module by considering two main issues. One is the variety of microseismic signals, and the other one is the nonlinearity of a microseismic signal. To extract different specific features in different frequencies and mathematical morphologies, multiple channels of kernels in different sizes should be used in each layer of the convolution module [32]. Differently sized kernels can catch differently scaled features, so the smallest size kernels are designed to capture the features in the highest frequency. A different channel of kernels is used to sense different features in a mathematical morphology. According to the successful examples using multiple-scale analyses in a microseismic signal and its sampling frequency, the parameters of these kernels are described in Figure 5. Because of the variety of microseismic signals, it is really hard to decide how to select the most effective kernels at each layer before obtaining some prior data, such as shoot data. So, a structure, such as “Inception”, is used in the convolution module, which applies a wide structure to let the convolution module decide which kernel to be used [33]. To increase the representational power of nonlinearity, a kind of structure, named “Network in Network” (NiN) [34], is used in the convolution module too. The NiN replaces filters with a micro-network compared with classical CNNs. Initially, the micro-networks are designed as fully connected multilayer perception, which actually increases the network’s depth. However, owing to information about the input or gradient passing through these layers, some of the captured characters may vanish when they reach the end or beginning of the network. So, we apply a densely connected structure used in DensNet [35], which creates short paths from early to later layers. Specifically, in a micro-network, each layer obtains additional inputs from all preceding layers and passes on its own feature-maps to all subsequent layers. Besides, according to the sparsity of microseismic data, the activation function in the convolution module is a rectifier linear unit, since a rectify activation function performs better when the data is sparse compared to sigmoid and hyperbolic tangent neurons. As the convolution module is designed to extract local features and to be easily implemented in an edge component, there are three convolution layers in the convolution module. Finally, the convolution module is designed as shown in Figure 5.

#### 3.2.4. RNN Module

As emphasized earlier, any microseismic event needs to be detected by considering temporal dependency as well. So, on the convolution module, there is a recurrent module in the neural network. However, there is the issue of a long-term dependency problem in microseismic detection when a microseismic event with a large energy takes place. If the energy of the microseismic event is large, the signal containing microseismic information will last a long time. So, detecting the end of a microseismic event is a problem, since it depends on some character of the arrival, which occurs a long time before the event’s end.

**Figure 6:**
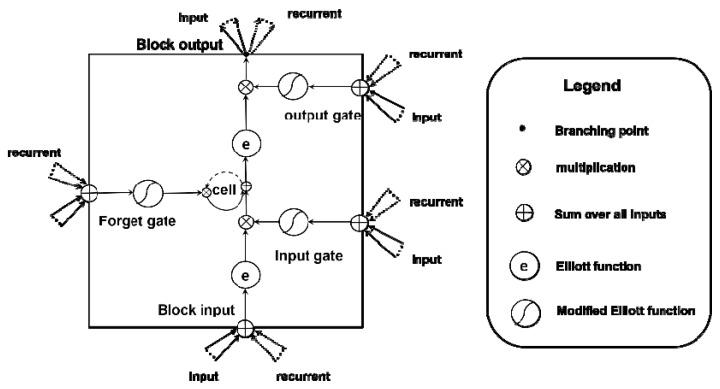
The architecture of LSTM.

Nowadays, the LSTM algorithm is well-known to be capable of learning the long-term dependencies problem [36]. The key property of LSTM is that it explicitly takes account of long-term information in the past which is a limitation of the classic RNN architecture (the long-term dependency problem). Considering former studies on LSTM, none of the variants can improve upon the standard LSTM architecture significantly [37], and activation functions are the most critical components of it [38]. So, in our study, we implement the standard LSTM architecture in the recurrent module and choose the Elliott function as the activation functions by considering the prior research on activation functions and the complexity of the neural network model’s implementation in an edge component. Instead of using sigmoid and hyperbolic tangent functions as activation functions, the Elliott-function-based LSTM can improve the detection accuracy without increasing any computational costs. Specifically, the activation functions of the input and output are the Elliott function showed as (1), while a kind of modified Elliott function is given in (2) that is applied as the activation function in the forget gate, input gate, and output gate. The architecture of the LSTM algorithm used in this paper is illustrated in Figure 6, and the related formulas are expressed from (3) to (7). To extract the temporal correlation of local microseismic features clearly, each LSTM cell connects to a feature map generated by the convolution module. In total, there are 72 feature map outputs generated by the convolution module. So, the cell number of every LSTM unit is 72.

(1) $$f(x)=\frac{x}{1+\left|x\right|}$$

(2) $$f(x)=\frac{0.5x}{1+\left|x\right|}+0.5$$

(3) $$i_{t}=\sigma_{i}(x_{t}\cdot W_{xi}+h_{t-1}\cdot W_{hi}+b_{i})$$

(4) $$f_{t}=\sigma_{f}(x_{t}\cdot W_{xf}+h_{t-1}\cdot W_{hf}+b_{f})$$

(5) $$C_{t}=f_{t}*C_{t-1}+i_{t}*\sigma_{C}(x_{t}\cdot W_{xC}+h_{t-1}\cdot W_{hC}+b_{C})$$

(6) $$O_{t}=\sigma_{o}(x_{t}\cdot W_{xo}+h_{t-1}\cdot W_{ho}+b_{o})$$

(7) $$H_{t}=o_{t}*\sigma_{h}(C_{t})$$

The output vectors from all LSTM units are fully connected to a softmax layer to describe the raw data’s likelihood of being included by a microseismic event.

### 3.3. Edge Component in ELMMP

#### 3.3.1. Overview of Edge Computing in ELMMP

The edge component of our platform accomplishes microseismic event detection and related data transmission. To improve the efficiency of the microseismic monitoring system, there are more computing tasks assigned to an edge component besides the ordinary works, such as data recording and transmitting, that are seen in the usual edge devices. The computing tasks in our edge component can be sorted into three major parts. The first computing task in the edge component is to reduce the noise of microseismic data in a real-time way. This part is mainly focused on reducing the noise caused by some changes in the environment, such as a human walking, a vehicle running, or just some varieties of wind, in order to increase the accuracy of microseismic event detection. The second one, following environmental noise reduction, in the edge component is to execute the trained neural network to calculate how the collected data is likely to be contained in a microseismic event. The trained neural network parameters are downloaded to the edge component from the data center once the training model reaches a relatively high and stable classification result. Then, the neural network is implemented in the edge component to provide the microseismic data probability. The last computing task is to infer whether each sampling point belongs to a microseismic event based on its probability given by the neural network model. Finally, only the data included by the microseismic event is sent to the data center, which is much less than the raw data recorded by the edge component.

#### 3.3.2. Noise Reduction of Microseismic Data

Obviously, there are lots of changes in the field during hydraulic fracturing. Changes caused both by humans and nature will induce environmental noise in the microseismic signal recording as long as they are close to the edge component. There is no doubt that a considerable amount of environmental noise is contained in a raw recording. To handle this issue, some filters are designed in an edge component for increasing the signal-to-noise ratio (SNR) of the microseismic data, which is critical to both microseismic event detection and related data analyses. Specifically, after the 24-bit analog-to-digital conversion, the raw data is post-processed by filtering with a series of Butterworth zero-phase, 2nd-order filters in the definite band-stop frequency. The start and stop frequency of the band-stop filters are defined according to the shoot data, since the shoot data contains the geophysical information of the hydraulic fracturing monitoring region and has common features with the acquisition data in the frequency domain.

#### 3.3.3. Implementation of the Neural Network

After the first computing task, the raw data is put into the trained neural network. To implement the trained neural network in an edge component when considering limited resources and the specific computing operations of our neural network, we use a parallel architecture. Different neural network structures have different computing operations; for example, CNNs’ main operations are mostly composed of spatial convolutions and the LSTM’s are matrix-vector multiplications. Since the proposed neural network model has two parts, namely convolution modules and LSTM modules, there are two strategies applied in the design of an edge component’s architecture.

**Figure 7:**
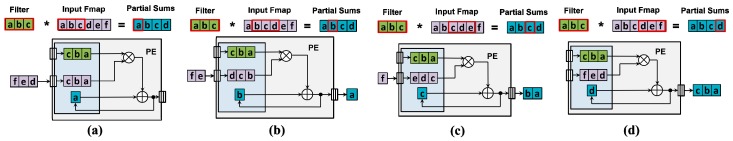
The convolutional computation reuse within a processing element (PE). (**a**) Step 1; (**b**) Step 2; (**c**) Step 3; (**d**) Step 4.

First, for the convolution module, the feature map of every layer is reused to reduce the computing load, and each convolution layer runs different-kernel convolutions in parallel by using multiply-accumulate units. The computation architecture of the convolution layer is shown in Figure 7. Specifically, for each kernel, the filter weights are stationary inside the register file (RF) of the processing element (PE) and the input feature is streamed into the PE. The PEs are used to finish the multiply-and-accumulate (MAC) operations for each sliding window at one time. Since there are overlaps of input features between two consecutive sliding windows, the input features can be kept in the RF and reused.

**Figure 8:**
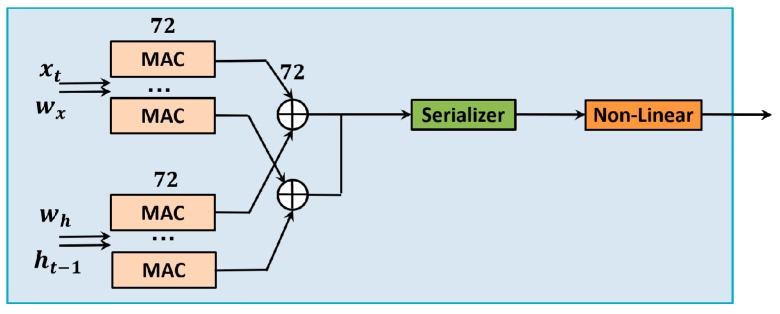
The structure of the gates block.

Secondly, for the LSTM module, many multiply-accumulate units are used here to accelerate the computing speed, since 71% of the run-time of an RNN is due to matrix-vector multiplication according to related work [39,40]. Hence, it is wise to use as many multiply-accumulate units as possible so that those operations can be processed simultaneously. In our work, the LSTM is one layer with 72 hidden cells. There are 144 multiply-accumulate units used in the gates block to accomplish matrix-vector multiplication as shown in Figure 8. One element of the input vectors (the output of the CNN module) and one element of all weight matrix rows are fed to 72 multiply-accumulate units to compute the matrix-vector multiplication. The other 72 multiply-accumulate units are fed by one element of the last state output vectors and one element of all weight matrix rows to compute the matrix-vector multiplication. Then, the results from the two groups of multiply-accumulate units are added together in parallel. These results, in a bus of data, are then serialized into a stream to compute the non-linear function (Elliott function or modified Elliott function). The non-linear function is an element-wise operation; thus, there is not much advantage to doing a parallel computation for non-linear mapping. Besides, there are weight and vector caches to store the parameters, and DMA ports are used to update the parameters.

**Figure 9:**
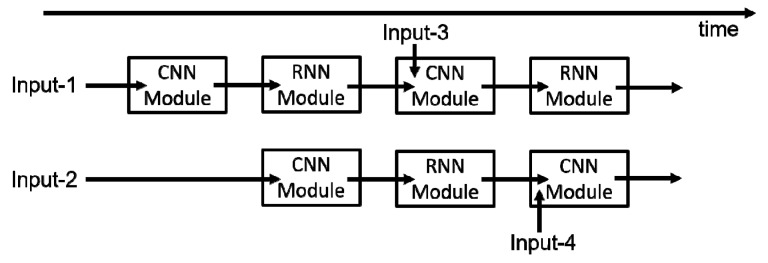
The computing architecture of the neural network in edge components. RNN, recurrent neural network.

For the whole neural network, its CNN module and RNN module are implanted in parallel rather than sequence to accelerate calculation. Specifically, the two modules are executed in a two-stage pipeline, and they are processed according to their related strategies mentioned above, respectively. The whole computing architecture of the neural network in an edge component is shown in Figure 9.

#### 3.3.4. Probabilistic Inference Module

Usually, neural networks are used as a kind of classifier to obtain discrete classification results directly. However, in a microseismic application, there is a strong dependence in consecutive time series data, where it is impossible to have many drastic fluctuations in classification results during only a few time intervals. Although the LSTM algorithm could capture some dependence features in the time domain by mining the series data, the output of the LSTM algorithm still has some drastic fluctuations if we let the LSTM algorithm generate discrete classification results directly. This kind of result cannot be considered as a final classification decision because outputs from LSTM are viewed independently without unambiguous probabilistic information of the classification. This problem may be caused by many different reasons, such as higher noise levels and a different pattern in microseismic events. According to these concerns, it is hard to only use neural networks and obtain high accuracy. Therefore, in this work, a probabilistic inference-based approach is used to handle this problem, which takes into consideration of the outcome from the neural network model. To decrease false negative errors in the classification results, as well as make the best use of the neural network model’s prediction, the output of the neural network model is considered as a microseismic event probability at each sampling data. Then, the probability is used to calculate the final classification result by using the following equations from (8) to (12). In the following equations, *x_i_* is the *i*-th sampling data, while *p*(*x_i_*) is the neural network’s output of the *i*-th sampling data, which can also be thought of as the probability to be a microseismic event in our work. Pme_i1_ means the degree of membership of the *i*-th sampling data to be a part of a microseismic event, while *Pme_i_*_0_ means the degree of membership of the *i*-th sampling data not to be included by any microseismic event. *Ptr_i_*_1_ represents the transition probability of the *i*-th sampling data to be a part of a microseismic event if the (*i* − 1)-th sampling data is not classified to be the label “1”. In the same way, *Ptr_i_*_0_ stands for the transition probability of the *i*-th sampling data to be sorted to class “0” if the (*i* − 1)-th sampling data is labeled “1”. *C*(*x_i_*) is the final result of the *i*-th sampling data. For the *i*-th sampling data, if its transition probability is larger than the membership value of the former sampling data, it will be sorted to a different class from the former one. Otherwise, it will be sorted to the same class as the former one, as (12) describes.

(8) $$Pme_{i1}=p(x_{i})$$

(9) $$Pme_{i0}=1-p(x_{i})$$

(10) $$Ptr_{i1}=p(x_{i})*p(x_{i+1})*p(x_{i+2})$$

(11) $$Ptr_{i0}=(1-p(x_{i}))*(1-p(x_{i+1})*(1-p(x_{i+2}))$$

(12) $$C(x_{i})=\{\begin{array}{c} 1，Ptr_{i1}>Pme_{i0}\\ 0，Ptr_{i0}>Pme_{i1} \end{array}$$

## 4. Evaluation

### 4.1. Simulation Results and Analysis

To evaluate the performance of our platform, we first conducted a series of simulation experiments. In the simulation, several Ricker wavelets with different frequencies, maximum amplitudes, and noise levels were used to evaluate the detection accuracy of our proposed algorithm, while the STA/LTA algorithm was also used as a benchmark for performance comparison.

First, 10 training sets were generated for the simulations and each training set contained 40-channel simulation microseismic data. Considering the main frequency band of the microseismic wave recorded on the surface, the simulations focused on the frequency band from 20 to 300 Hz. In each simulated microseismic channel, several Ricker wavelets within a frequency band from 20 to 300 Hz were generated, each of which consists of a time series considering the characteristics of real microseismic data in the frequency domain. Then, in the generated series, the data that consists of Ricker wavelets was labeled with “1”. Otherwise, the data between two adjacent Ricker wavelets in the series was labeled as “0”. Finally, the series data and its labels were used to train our neural network.

To ensure good reliability, a testing set with 40 channels was generated. Each channel includes six Ricker wavelets, whose frequencies were randomly chosen from 20 to 300 Hz. In the testing set, there was a Ricker wavelet with the particular domain frequency in every 200 samples. So, each channel has 1200 samples. Then, these 40 channels of testing data were added with different levels of Gaussian White Noise. Specifically, the signal-to-noise ratio (SNR) of the test data were 0 dB, −5 dB, −10 dB, and −15 dB, respectively.

**Figure 10:**
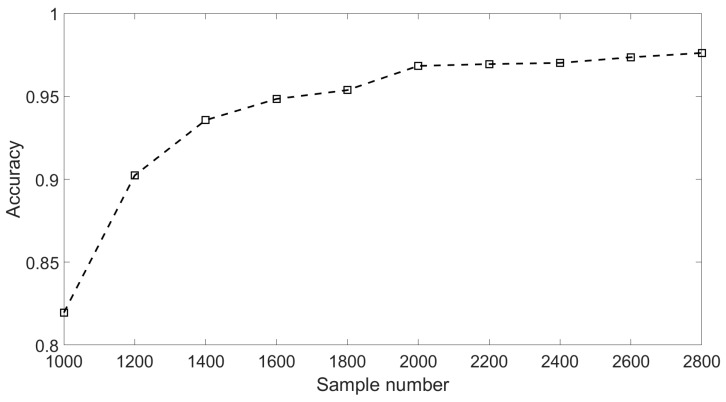
Results of different sample numbers.

To illustrate the relationship between the number of training samples and the model’s prediction accuracy, all of the 10 training sets were involved in the simulations. Each training set had 40 channels. To be specific, in each channel, a Ricker wavelet with a particular domain frequency was included in every 200 samples. In order to illustrate the impact of different sample numbers conveniently, the wavelet number and sample number of each channel in the same training set were set to be an equal length. In the 10 different training sets, the number of Ricker wavelets contained by each channel ranged from 5 to 14, respectively. As a result, the total sample numbers of each channel in the 10 different training sets ranged from 1000 to 2800. Then, the 10 different training sets were used to train the model. After training, the models were tested by the data with an SNR of −15 dB in the testing set. The testing results are compared in Figure 10. According to the results shown in Figure 10, it could be figured out that the training set including 10 Ricker wavelets (containing 2000 samples) is able to enable the model to achieve an acceptable and stable accuracy of over 96%.

**Figure 11:**
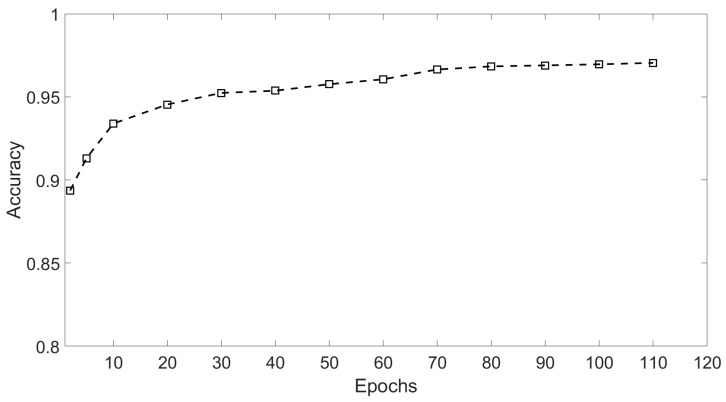
Results of different epochs.

In addition, to give a reasonable result of the training epochs needed by the model to reach a reliable detection, we had also compared the different classification accuracies achieved by the model with different training epochs. To be specific, the 10 Ricker wavelets (2000 samples) were used to train the model. The data with an SNR of −15 dB in the testing set was selected as testing data. Then, the trained models with 13 different epochs were tested, respectively. The relationship between the classification accuracy and training epochs in the simulation is shown in Figure 11. After 80 epochs, the model achieves a relatively high and stable classification result.

**Figure 12:**
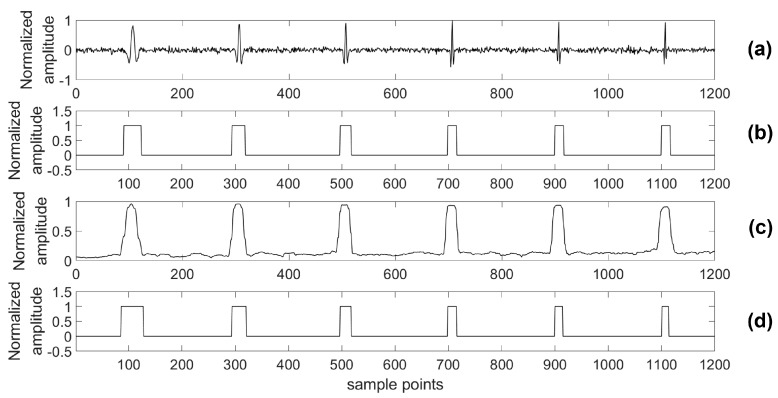
Channel A of test data (0 dB) and its results. (**a**) Test data (0 dB); (**b**) Detection results of the proposed algorithm; (**c**) Detection results of the short-term average to long-term average (STA/LTA) algorithm; (**d**) The real label.

**Figure 13:**
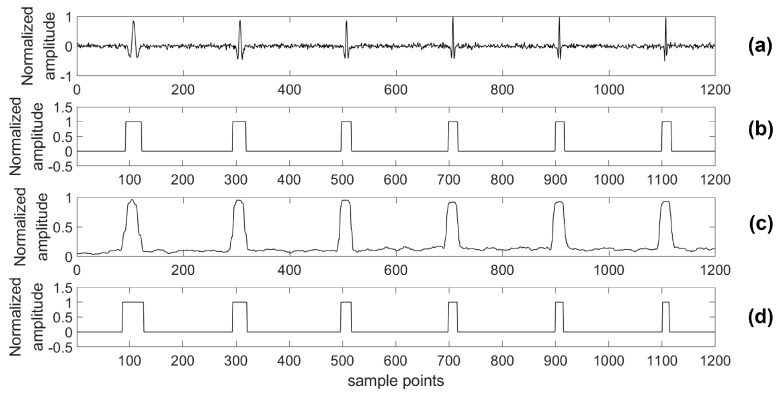
Channel B of test data (0 dB) and its results. (**a**) Test data (0 dB); (**b**) Detection results of the proposed algorithm; (**c**) Detection results of the STA/LTA algorithm; (**d**) The real label.

**Figure 14:**
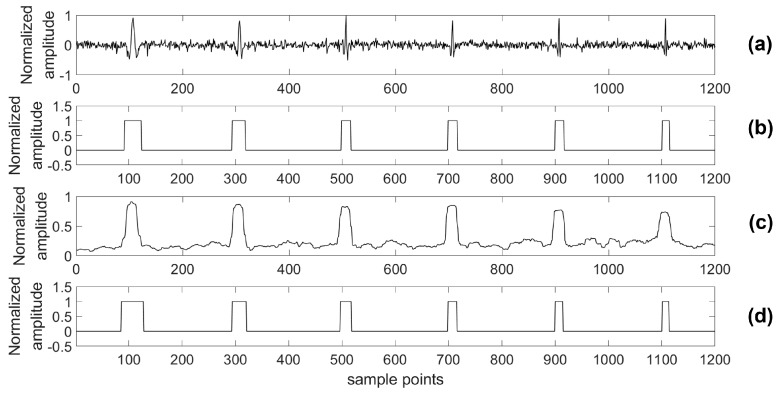
Channel A of test data (−5 dB) and its results. (**a**) Test data (−5 dB); (**b**) Detection results of the proposed algorithm; (**c**) Detection results of the STA/LTA algorithm; (**d**) The real label.

**Figure 15:**
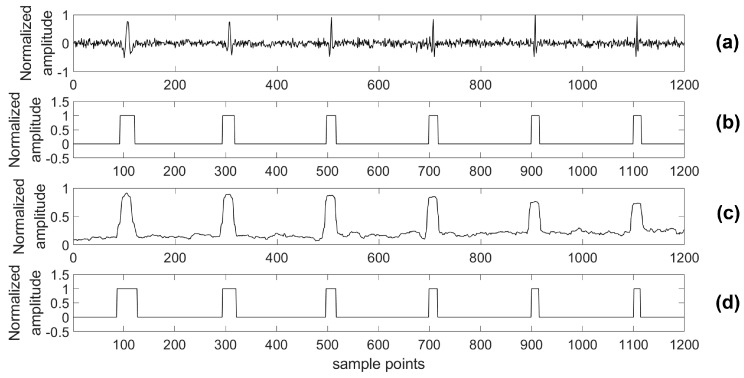
Channel B of test data (−5 dB) and its results. (**a**) Test data (−5 dB); (**b**) Detection results of the proposed algorithm; (**c**) Detection results of the STA/LTA algorithm; (**d**) The real label.

**Figure 16:**
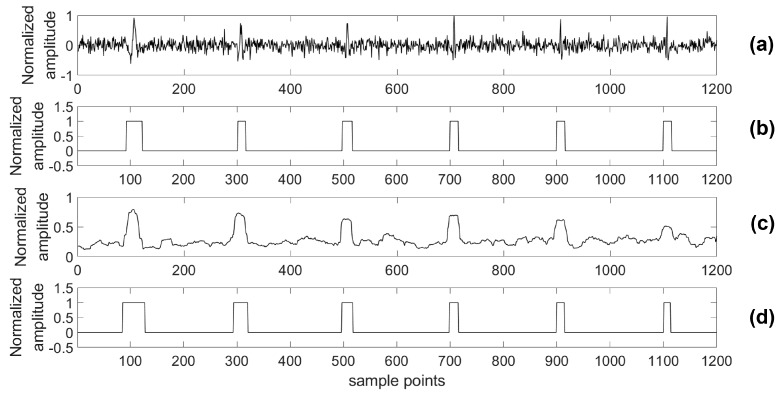
Channel A of test data (−10 dB) and its results. (**a**) Test data (−10 dB); (**b**) Detection results of the proposed algorithm; (**c**) Detection results of the STA/LTA algorithm; (**d**) The real label.

**Figure 17:**
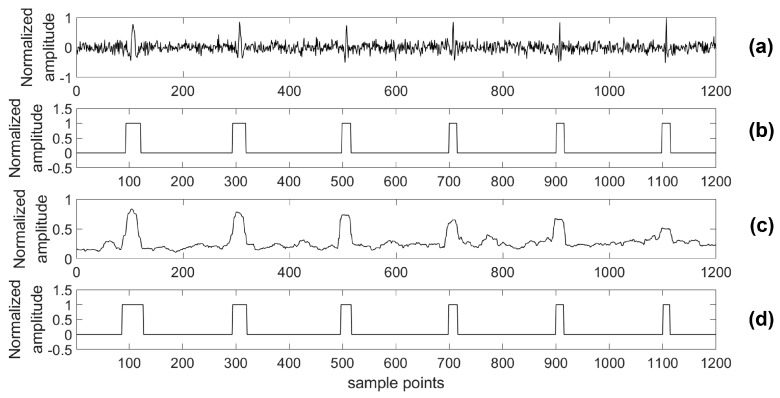
Channel B of test data (−10 dB) and its results. (**a**) Test data (−10 dB); (**b**) Detection results of the proposed algorithm; (**c**) Detection results of the STA/LTA algorithm; (**d**) The real label.

**Figure 18:**
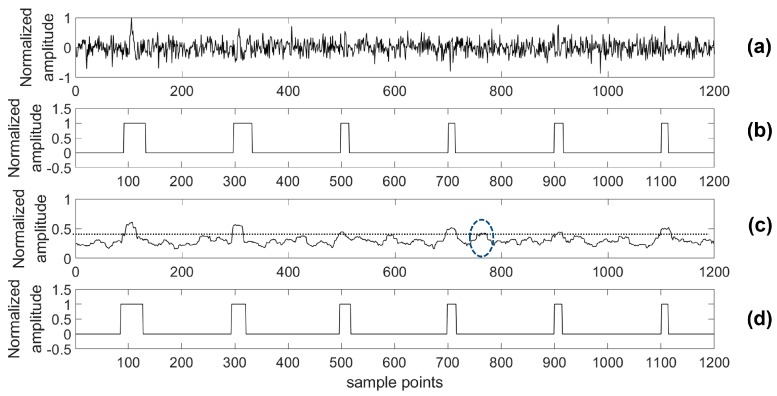
Channel A of test data (−15 dB) and its results. (**a**) Test data (−15 dB); (**b**) Detection results of the proposed algorithm; (**c**) Detection results of the STA/LTA algorithm; (**d**) The real label.

**Figure 19:**
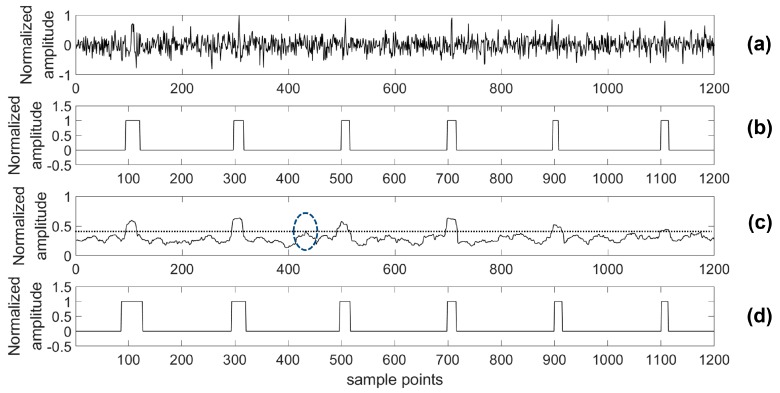
Channel B of test data (−15 dB) and its results. (**a**) Test data (−15 dB); (**b**) Detection results of the proposed algorithm; (**c**) Detection results of the STA/LTA algorithm; (**d**) The real label.

For providing more sound shreds of evidence on robust performance, the proposed algorithm was compared with the STA/LTA algorithm for detecting a microseismic event in test series data with different SNRs. Particularly, considering the situation of a real application, a 4th-order bandpass filter was used prior to the STA/LTA algorithm, while the proposed algorithm was used to process the test data directly. As with the related training parameters provided above, the model was trained by the training set with 2000 samples in each channel as well as 80 training epochs. Two typical channels (A and B) of test data were chosen to illustrate the detection results of the two algorithms and the real labels are shown in Figure 12, Figure 13, Figure 14, Figure 15, Figure 16, Figure 17, Figure 18 and Figure 19.

Based on the results of this simulation, one of the advantages of our presented detection algorithm has been clearly shown. Using the presented algorithm, the impact on detection accuracy due to different noise levels is marginal compared with the STA/LTA-based approach. Especially, there is a definite advantage of the proposed algorithm in the low SNR case compared with the STA/LTA. For example, with an SNR of −15 dB, the proposed algorithm is able to detect every event. However, there is a mistake of taking noise as an event in the STA/LTA algorithm as shown by Figure 18 and Figure 19. Specifically, in Figure 18, the part highlighted by a circle was a false negative classification when the STA/LTA algorithm tried to detect the third or fifth event in the series data using a threshold, which is expressed as the dashed line. A similar situation could be found in the other channel as shown in Figure 19.

For detailed analysis, three metrics (accuracy, precision, and recall) of the two detection algorithms are calculated and the results are listed in Table 1. Note that, in order to get the best result of using the STA/LTA algorithm, the classification threshold has to be changed according to different noise levels. In fact, it is difficult to obtain an appropriate threshold for all cases in practice, since the SNR always changes in a random way. To be more persuasive, the proposed algorithm is compared with the best result of STA/LTA, whose thresholds are selected manually. Despite this, the proposed algorithm performs better than the STA/LTA algorithm in terms of all of the three metrics, especially in a high-level noise situation. Besides this, considering the results of precision and recall, there is another advantage of the proposed algorithm, which is that it is hardly affected by the imbalanced data. Furthermore, although both the proposed algorithm and the STA/LTA algorithm seemed to well-detect events with high SNR data, the classification quality is different. Detection with higher quality means less redundancy in the data to be transmitted to the data center, which leads to a more efficient system. Therefore, the proposed algorithm also enables the platform to be more efficient compared with the STA/LTA algorithm.

To illustrate the proposed algorithm’s performance on data reduction, the ratio of data reduced is calculated and its results are shown in Table 2. The ratios of data reduced among different SNRs are about 90%. That means that the data transmission time will be saved by 90% and the real-time performance of the microseismic monitoring system will be definitely improved.

### 4.2. Measurement Results and Analysis

The proposed monitoring platform has also been applied in a testing microseismic monitoring system, which is researched by Jilin University [10,41]. The testing monitoring system includes surface monitoring units, borehole monitoring units, access points, and a data center. The surface monitoring units and borehole monitoring units are thought of as edge components, which are used to collect the microseismic data and transport data to the data center. The monitoring units are implemented by Xilinx’s Field-Programmable Gate Array (FPGA), while the data center is based on an Intel Server in a vehicle. The whole testing microseismic monitoring system with our proposed platform was used to measure real microseismic data in the northeastern China during a hydraulic fracturing project. During hydraulic fracturing monitoring, there were 36 edge components applied to the surface. Each edge component was connected to a three-axis sensor, which means that the total number of channels used in this measurement was 108. The 108-channel shoot data in that project were used to train the neural network in the proposed detecting algorithm, and there were 1400 samples in each channel. We labeled this training set according to the amplitude of the microseismic record and the shoot time. One channel of the training data and its corresponding labels are shown in Figure 4.

**Figure 20:**
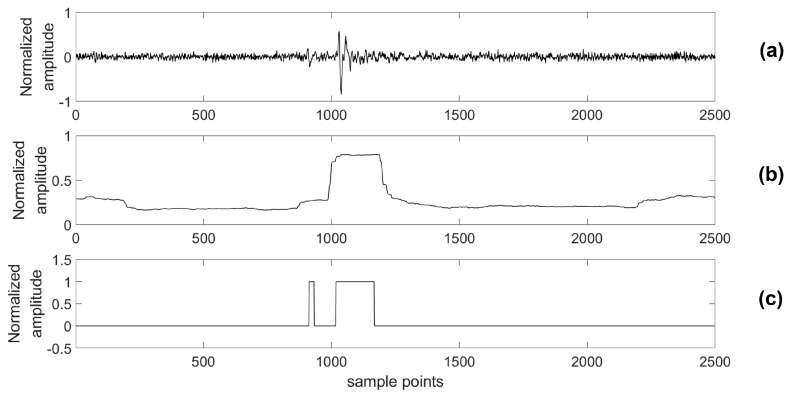
Original data and its classification results. (**a**) The original data; (**b**) The classification results of the STA/LTA algorithm; (**c**) The classification results of proposed algorithm.

**Figure 21:**
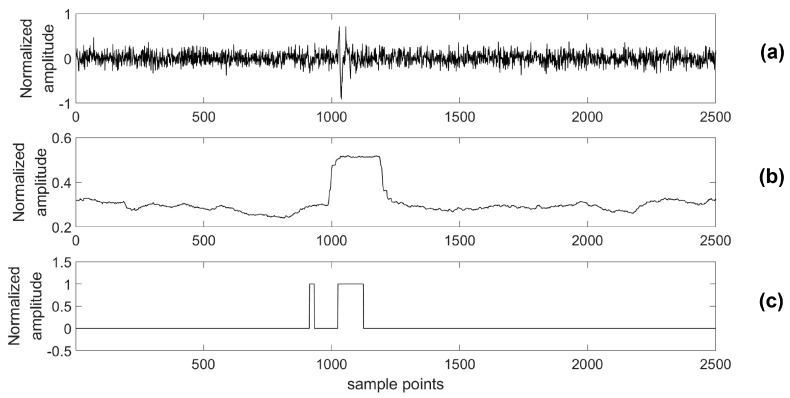
The data with extra noise and its classification results. (**a**) The data with extra noise; (**b**) The classification results of the STA/LTA algorithm; (**c**) The classification results of the proposed algorithm.

A testing data set was generated with 108 channels; each channel included 60,000 samples. To verify that our platform has the capability to detect a microseismic event with high accuracy even in a low SNR situation, we added additional Gaussian White Noise, whose energy is 3 times larger than that of the original signal, to real data sensed by the testing system. Then, both the original data and the data with added noise were processed by the proposed detecting algorithm and the STA/LTA algorithm, respectively. Similar to the simulations, a 4th-order bandpass filter was also used prior to the STA/LTA algorithm. One piece of these data and its classification results are shown in Figure 20 and Figure 21.

In practical cases, it is usual for microseismic data to be consecutive microseismic events with different energies. In Figure 20, two microseismic events with different energies are detected clearly by the proposed algorithm, while the microseismic event with lower energy is missed by the STA/LTA algorithm. This result shows that it is easy for the STA/LTA algorithm to miss some low-energy events that are very close to a microseismic event with larger energy, while the proposed algorithm can definitely detect this type of event with high quality. In the low SNR case, shown by Figure 21, the proposed algorithm is capable of detecting the lower energy microseismic event even though it is drowning in noise and can be hardly recognized by a human. Therefore, compared with the STA/LTA algorithm, the measurement results prove that the algorithm used in our platform has an advantage in detecting multiple events that take place in a very short time interval even in a low SNR situation.

In this segment of measurement data, the ratio of transmitted data reduced is 93.1%. So, with the proposed platform, this testing microseismic monitoring system transmits data in a more efficient way, and its performance on real-time data collection can be improved obviously.

## 5. Conclusions

In this work, the design of a new Edge-to-Center LearnReduce Microseismic Monitoring platform is presented, which consists of edge components and a data center. In this platform, an edge component is used for not only data transmission but also event detection. Since microseismic signals and noise have different properties, a new event detection algorithm based on a neural network and a probability inference is presented for edge computing. The proposed method is tested by both synthetic and measured microseismic signals. Through comparison with the STA/LTA method, the proposed method was found to improve the detection accuracy significantly even when the noise is strong. Moreover, after detection, the volume of data needed to be transmitted to the data center could be reduced by about 90%. Therefore, according to the detection accuracy and the ratio of transmission data reduced, we could conclude that the presented platform is able to improve the efficiency of real-time microseismic monitoring, which means it has great potential to be used in practice.

## Figures and Tables

**Table 1:** The contrast of detection accuracy.

SNR (dB)	STA/LTA Threshold	STA/LTA Accuracy (%)	STA/LTA Precision (%)	STA/LTA Recall (%)	Proposed Algorithm Accuracy (%)	Proposed Algorithm Precision (%)	Proposed Algorithm Recall (%)
0	0.41	96.92	89.63	84.72	98.58	94.07	93.38
−5	0.37	96.85	88.14	82.76	98.42	93.35	92.64
−10	0.38	96.78	86.67	81.81	97.45	89.72	88.53
−15	0.39	95.25	80.13	78.26	96.83	87.36	86.12

SNR, signal-to-noise ratio; STA/LTA, short-term average to long-term average.

**Table 2:** The ratio of data reduced in different SNRs.

SNR (dB)	Ratio of Data Reduced (%)
0	89.17
−5	89.67
−10	90.67
−15	88.75
